# Exploring spatial patterns of acupoint indications from clinical data: A STROBE-compliant article

**DOI:** 10.1097/MD.0000000000006768

**Published:** 2017-04-28

**Authors:** Won-Mo Jung, Soon-Ho Lee, Ye-Seul Lee, Younbyoung Chae

**Affiliations:** Acupuncture and Meridian Science Research Center, College of Korean Medicine, Kyung Hee University, Seoul, Republic of Korea.

**Keywords:** acupuncture, indication, meridian system, spatial information, statistical parameter

## Abstract

Every acupoint has specific indications for acupuncture treatment. These indications, primarily established based on the meridian system, have spatial patterns of symptoms on the human body. We investigated the associations between acupoints and symptom locations in 75 patients with chronic pain who were asked to sketch the localization of their symptoms on body schemes using the bodily sensation map (BSM) system. Combining the BSM and clinical information, we estimated the statistical parameters of relationships between acupoints and spatial information on symptoms. We further visualized spatial patterns of indications of the representative acupoints on the human body template using a Z score. Using a statistical parametric map method, we observed significant activation patterns of 12 acupoint indications with spatial patterns. The 1st group of patterns was distant from the acupoint locus and was strongly associated with the route of the corresponding meridian. The 2nd group was found around the acupoint locus, the majority of which was located at the trunk or back areas. Intensive investigations of the spatial patterns of acupoint indications would be a novel paradigm to explain point specificity of acupuncture treatment based on the original concept of the meridian system. Future studies should include more meaningful clinical data with larger sample sizes.

## 1 Introduction

Each acupoint has a wide assortment of indications, and conversely, each disease or indication can be treated by a wide variety of acupoints.^[1,2]^ Wang et al^[3]^ demonstrated that the selection of acupoints for every symptom and the matching of symptoms for every acupoint showed a different distribution pattern. Thus, there is an urgent need to standardize acupoint indications.^[4]^ Many studies have made great efforts to explore point specificity in acupuncture treatment according to acupoint indications.^[5–7]^ Importantly, these indications, originally established based on the meridian system, have spatial patterns of symptoms on the human body.^[8]^

Classic medical textbooks on acupuncture and moxibustion state that major acupoints, particularly those below the knees and elbows, have both regional and remote effects across the entire body. The meridian system, as an ancient infographic method, represents constellations of acupoints that have common therapeutic actions.^[9]^ Because the lines of the meridian system are highly associated with the acupoint indications, understanding the meridian system could provide us information regarding acupoints that are better for the treatment of a particular disease or a set of symptoms.^[10,11]^ Using text-mining methods of classic medical texts, we previously showed that spatial patterns of the indications of acupoints, particularly below the knees and elbows, are strongly associated with the route of the corresponding meridian.^[8]^ However, there have been no studies on the relationship between acupoints and the locations of symptoms on the body based on clinical data.

There are a large number of well-validated measures for the intensity and characteristics of pain.^[12]^ However, it is also important to identify the most painful areas. When we consider the relationship between acupoints and symptoms, it is important to measure areas that are painful on the body. Recently, Beissner and Marzolff^[13]^ used a geographic information system and proposed that acupuncture-evoked sensations that propagate along channels are closely associated with classic meridians. Recently, we developed a bodily sensation map (BSM) system to measure and analyze spatial information of painful symptoms across the entire body.^[14]^

In the present study, we recruited chronic pain patients treated with acupuncture and created a BSM to gather spatial information on their symptoms. We also visualized the spatial patterns of indications of the representative acupoints based on statistical parameters of the indications related to spatial information.

## 2 Methods

### 2.1 Participants

A total of 75 patients (females, 49; age, 61.8 ± 16.4) took part in the study. All patients were recruited at a traditional Korean medical clinic in the Republic of Korea. To control symptoms and treatment, only patients who visited for the treatment of chronic pain by acupuncture were included (no restrictions on the location of pain). One male Korean medical doctor participated in this study. The patients were informed on the nature of the experiment and provided full written consents. The study was conducted in accordance with the Declaration of Helsinki and approved by the Institutional Review Board of Kyung Hee University in Seoul, Republic of Korea.

### 2.2 Study design and procedure

**Figure 1:**
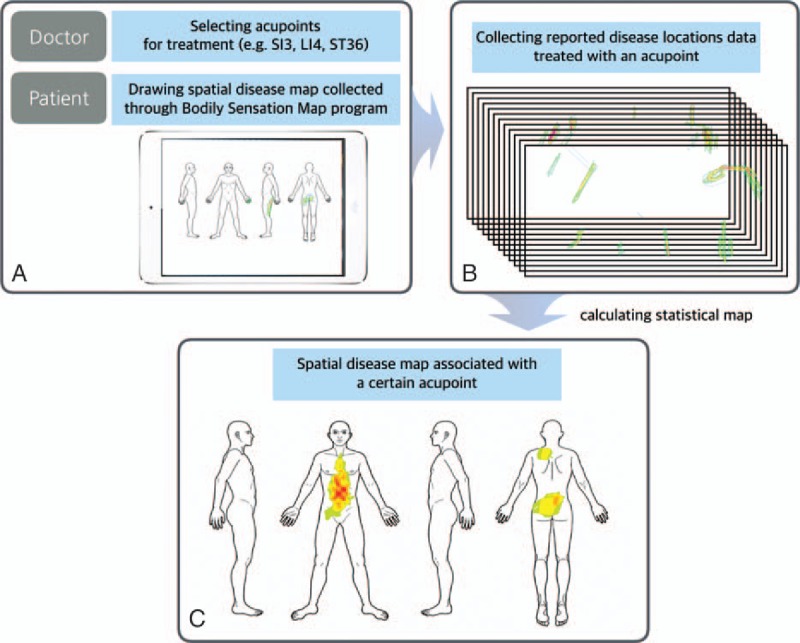
Data collection, analysis, and visualization. (A) A human body template was shown as a 2D image in 4 view angles (front, back, left, and right side view) using the bodily sensation map (BSM) system. Patients were required to draw their symptom sites onto the BSM interface using a touch pen. (B) Then we estimated the associations between acupoints and the spatial information of symptoms by combining the BSM data and clinical information. (C) We visualized spatial patterns of acupoint indications on the human body template using Z scores.

After initial diagnosis of the acupuncture treatment, the doctor asked patients to sketch the localization of their symptoms on the body schemes using the BSM.^[14]^ In the BSM, a human body template is presented as a 2D image, shown from 4 different angles (front, back, and 2 lateral views) (Download this image and the program from the website: http://cmslab.khu.ac.kr/downloads/bsm). Successive strokes on a region with a touch pen changes the color of that area. Our participants were asked to do this, and the resulting drawings were collected in 2048 × 1024 matrices using an Apple iPad mini (Fig. 1).

The doctor made decisions for acupuncture treatment based on the initial diagnosis and information on the locations of symptoms.

### 2.3 Data analysis

We recorded the baseline characteristics of acupoint information from all patients for the treatment of chronic pain. Because spatial patterns from acupoints that are not commonly used in clinics are most likely exaggerated or distorted, we included only the top 20 most common acupoints among 60 acupoints used. Among the 20 acupoints, we further explored those with a significant amount of information on their spatial patterns.

Combining the BSM with clinical information, we estimated the relationships between acupoints and the spatial patterns of symptoms. Then we visualized the patterns on the human body template using Z scores.

Prior to group-level analysis, individual data from each patient was scaled into a range between 0 and 1. Random effects analysis and pixel-wise univariate *t* tests were performed on a group-level spatial pattern map within a mask of used body template (3dttest, AFNI, http://afni.nimh.gov/afni), and the *t* value maps were transformed into Z-scores. Correction for multiple comparisons was performed by running 10,000 Monte Carlo simulations using the AFNI AlphaSim program.^[15]^ The width of the touch brush (15 pixels) used to draw spatial disease maps was entered as the extent of spatial correlation (smoothness). Activation patterns were defined as corrected significance level (alpha level) of 0.05 (with cluster size >1704 pixels). In the statistical maps, the Z-scores of pixels reflected the spatial information of acupoint indications for treatment.

## 3 Results

### 3.1 Baseline characteristics of used acupoints and 2 different activation patterns

A total of 60 acupoints were chosen for the treatment of 75 patients with chronic pain. Some of the most common acupoints included LI4 (37.3%), SI3 (29.3%), TE5 (20.0%), PC6 (20.0%), EX-B2 (20.0%), GB30 (17.3%), SP6 (14.7%), LI11 (14.7%), GB21 (14.7%), BL60 (14.7%), GB34 (13.3%), ST43 (13.3%), LI10 (12.0%), BL40 (10.7%), and CV12 (10.7%) (Table 1).

**Table 1:**
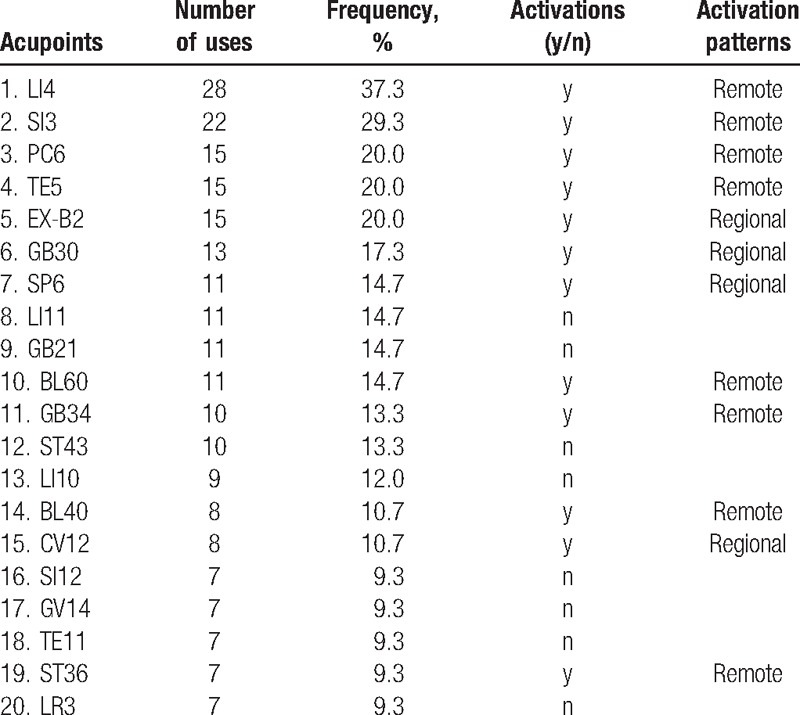
Baseline characteristics of acupoints and 2 different activation patterns.

Among the 20 acupoints, there were activation patterns for 12, which showed greater associations with spatial information. Furthermore, we divided the spatial maps into 2 activation patterns: regional (when they included the locus of the corresponding acupoint) and remote (all others). Eight acupoints (LI4, SI3, PC6, TE5, BL60, GB34, BL40, and ST36) had remote activation patterns, and 4 (EX-B2, GB30, SP6, and CV12) had regional patterns.

### 3.2 Indication maps of acupoints with remote activation patterns

**Figure 2:**
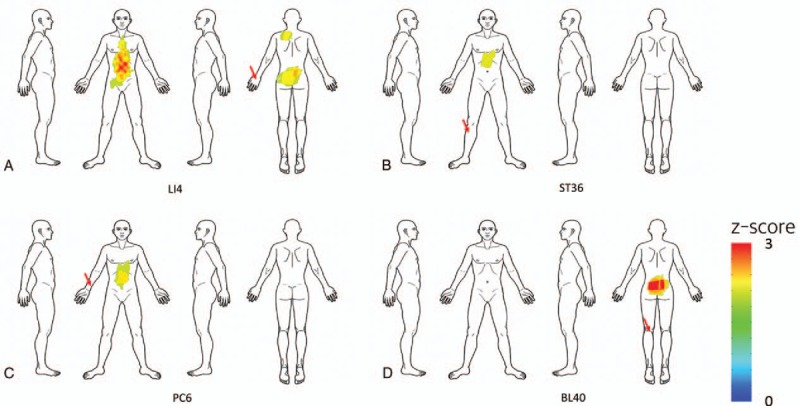
Indication maps of acupoints with remote activation patterns. We visualized spatial patterns of 4 representative acupoints with remote activation patterns: (A) LI4, (B) ST36, (C) PC6, and (D) BL40. Each acupoint showed a distinct spatial pattern of indications and was strongly associated with the route of the corresponding meridian. Red arrows indicate the corresponding acupoint locations in the body.

We visualized the spatial patterns of 4 representative acupoints with remote activation patterns. Each one had a distinct spatial pattern of indications, and its activation patterns were relatively distant from its locus. The patterns were strongly associated with the route of the corresponding meridian (Fig. 2).

Acupoint LI4, in the large intestine meridian, showed stronger associations with the frontal area of the body, including the lower part of the abdominal region. ST36, in the stomach meridian, showed stronger associations with the frontal area of the body including the upper abdominal region. PC6, in the pericardium meridian, showed greater associations with the frontal area of the body including the chest and upper abdominal region. BL40, in the bladder meridian, showed greater associations with the back area of the body, including the lower back region.

### 3.3 Indication map of acupoints with regional activation patterns

**Figure 3:**
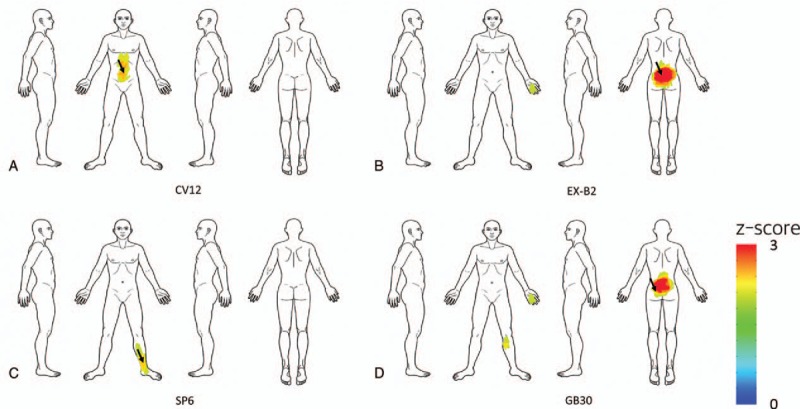
Indication maps of acupoints with regional control patterns. We visualized the spatial patterns of 4 representative acupoints with regional activation patterns: (A) CV12, (B) EX-B2, (C) SP6, and (D) GB30. Each acupoint had a relatively regional spatial pattern of indications. Most of them were located in the trunk and back areas. Black arrows indicate the corresponding acupoint locations in the body.

We also visualized the spatial patterns of the 4 acupoints with regional activation patterns. The majority of the spatial patterns were located in the trunk and back areas (Fig. 3).

CV12, in the conceptual vessel meridian, showed stronger associations with the abdominal area. GB30, in the gall bladder meridian, showed stronger associations with the lower back area.

### 3.4 Similar spatial patterns of 2 different acupoint indications

**Figure 4:**
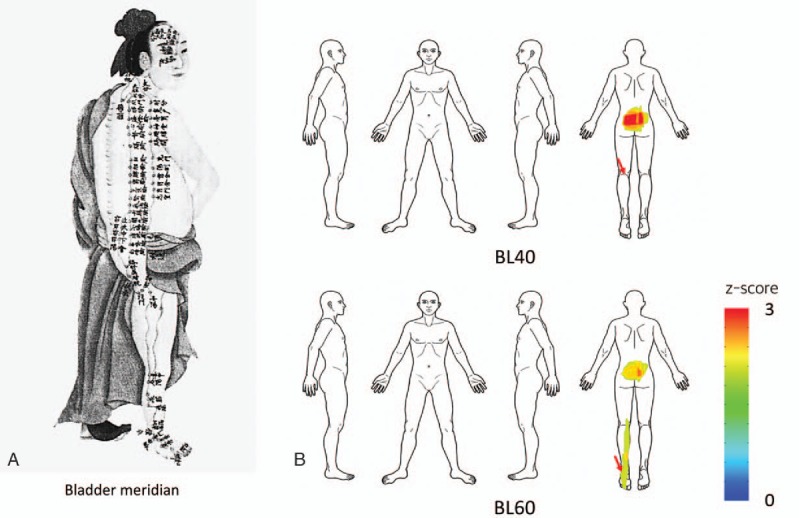
Comparison of Meridian diagram in Qing Dynasty and spatial patterns of acupoint indication. (A) A traditional Mingtang Diagram for the bladder meridian from the Qing Dynasty. (B) Acupoints BL40 and BL60 in the same meridian showed similar spatial patterns of indications.

BL40 and BL60, which were in the same meridian, showed similar spatial patterns of indications, with stronger associations with the dorsal area of the body, including the lower back region (Fig. 4). Their patterns were consistent with the route of the bladder meridian in the traditional Mingtang Diagram of the Qing Dynasty.

## 4 Discussion

Among the 20 most common acupoints, 12 had significant activation patterns of indications, which we divided into 2 groups: remote (far from locus) and regional (near the locus). Remote patterns were strongly associated with the routes of corresponding meridians. Regional ones were mostly located in the trunk or back area. This study is the first step to further examine spatial patterns of acupoint indications from real-world clinical data.

We visualized the spatial patterns of 4 representative acupoints that had remote activation patterns (Fig. 2). Each showed a distinct pattern of indications, and its activation patterns were distant from its locus. According to classic medical texts, acupoint indications are closely related to spatial information on symptoms.^[8]^ For example, acupoints LI4, ST36, and BL40 are used to treat the face, abdomen, and lower back, respectively. We found that ST36 had a greater association with the abdominal area, while BL40 had a greater association with the lower back area. Most of spatial patterns of indications were consistent with the indication information from bibliographical data. On the other hand, we identified 4 acupoints with regional activation patterns (Fig. 3). Most of them were located in the trunk and back areas, suggesting that most acupoints located in these areas show regional control effects.

Interestingly, BL40 and BL60, which were in the same meridian, showed similar spatial patterns of indications, particularly for the treatment of lower back pain (Fig. 4). The traditional Mingtang Diagram illustrates ancient infographics of the meridian system, for example, some acupoints in the bladder meridian could show remote activation of the posterior part of the body, including the lower back area. In a previous study, we visualized biomedical information on acupuncture treatment from a clinical trials database and found that BL40 and BL60 (used at a 32% frequency) were the most common acupoints for the treatment of lower back pain.^[9]^ In the present study, we visualized similar spatial patterns of the indications of these 2 acupoints by estimating the associations between each acupoint and symptom locations. We demonstrated the original concept of the meridian system based on clinical data in the real world.

Our previous study, based on text-mining methods of classic medical texts, showed that spatial patterns of acupoint indications on the body map were strongly associated with the route of the corresponding meridians.^[8]^ However, bibliographical data included only 18 disease sites on the body template, and no spatial location information. Using the BSM system, the present study provided more detailed information regarding spatial patterns of acupoint indications from a clinical database. Because this system had more accurate location information on symptoms from patients, it allowed us to extract more specific spatial information on the body. We believe that this system would be valuable for other pain studies investigating spatial information from the body template.

Some acupoints (particularly below the elbow and knee) showed different spatial patterns with remote pain-control effects, suggesting that acupuncture treatment has point-specificity. Because previous clinical trials have focused on the efficacy of acupuncture treatment by comparing real acupuncture treatment to placebo or sham treatment, it is difficult to identify the point-specificity of acupuncture treatment.^[16]^ Many studies have used neuroimaging methods to identify acupoint specificity by comparing neuronal responses to stimulation at acupoints to nonacupoint controls or other acupoints.^[17]^ Because there are greater commonalities across acupoints (ie, DeQi or pain-like sensations) in the central nervous system, such studies have failed to reveal differential brain changes in specific acupoints.^[5]^ To properly address the point-specificity issue, it is important to apply the original concept of the meridian system. Given that the meridian system proposes that there are a series of connections among different areas, organs, and functions, we demonstrate that each acupoint has different characteristics in acupuncture treatment in the clinic by visualizing spatial patterns of acupoint indications.

Visualizing acupoint indications based on bibliographical data can be used to show specific patterns similar to the corresponding meridians, providing a deeper understanding of meridians through acupoint indications and the symptoms that they treat. Furthermore, our study using clinical data provided consistent results, showing that acupoints used to treat chronic pain have both remote and regional control patterns. Previous studies have attempted to visualize meridians by matching trigger points and associated pain to acupoints, and have shown that acupuncture treatment can have remote effects on myofascial pain.^[18,19]^ Although acupoints and the meridian system, as well as myofascial pain and trigger point regions, show some similarities, our approach takes into account the meridian system as a group of treatment indications and symptoms, including chronic pain. Acupoints such as LI4 and ST36 showed remote activation effects in the abdominal area, surpassing the range of myofascial symptoms. Our approach of understanding acupoints through their indications and based on the meridian system through a group of similar treatment indications includes a wider range of symptoms. With the accumulation of clinical data, this approach can visualize the depth and range of clinical indications of the meridian system.

This study had several limitations. First, spatial patterns of acupoint indications were only extracted from 75 chronic pain patients treated by 1 doctor. Because most of the patients had lower back pain symptoms, it is possible that the spatial patterns of indications were slightly biased toward greater associations with the lower back region, regardless of the acupoints studied. To minimize distortions of the patterns of indications, it is important to perform the analysis with more data sources from greater sample sizes and with various doctors. Second, the indications were extracted from acupoints selected for the treatment of certain body areas, but did not reflect the degree to which these acupoints work. To provide more information on acupoint indications, this approach should be extended to include outcome information before and after acupuncture treatment. Finally, we used only spatial information from chronic pain patients and did not include other symptoms and/or cause information. When doctors select acupoints, they consider a wide range of symptoms. For example, some acupoints may ameliorate fever but lack any spatial pattern information. Thus, future studies are required to combine spatial information with information on other symptoms.

## 5 Conclusions

We explored the associations between acupoints and symptom locations on the body based on clinical data from real clinical practice, and visualized spatial patterns of indications. Using the BSM system, we found that each acupoint had a distinct spatial pattern of indications, and that the patterns were strongly associated with routes of the meridian. We propose that this system can be used to gather more meaningful clinical data with a larger sample size. We believe that our approach can be extended to reveal the point specificity of acupuncture treatment based on the meridian system.

## Acknowledgments

The authors thank Basic Science Research Program through the National Research Foundation of Korea (NRF) funded by the Ministry of Science, ICT & Future Planning (No. 2015R1D1A1A01058033) for the support.

## References

[1] Ha L Li T Wang F Exploration and analysis on the “similar-indication acupoints”. Zhongguo Zhen Jiu 2015;35:1263–5. 26964173

[2] Napadow V Liu J Kaptchuk TJ A systematic study of acupuncture practice: acupoint usage in an outpatient setting in Beijing, China. Complement Ther Med 2004;12:209–16. 15649834 10.1016/j.ctim.2004.10.001

[3] Wang YY Lin F Jiang ZL Pattern of acupoint selection based on complex network analysis technique. Zhongguo Zhen Jiu 2011;31:85–8. 21355168

[4] Huang LX Standard expression of indications of acupoints. Zhongguo Zhen Jiu 2007;27:823–7. 18085146

[5] Chae Y Chang DS Lee SH Inserting needles into the body: a meta-analysis of brain activity associated with acupuncture needle stimulation. J Pain 2013;14:215–22. 23395475 10.1016/j.jpain.2012.11.011

[6] Choi EM Jiang F Longhurst JC Point specificity in acupuncture. Chin Med 2012;7:4. 22373514 10.1186/1749-8546-7-4 PMC3311034

[7] Xing JJ Zeng BY Li J Acupuncture point specificity. Int Rev Neurobiol 2013;111:49–65. 24215917 10.1016/B978-0-12-411545-3.00003-1

[8] Jung WM Lee T Lee IS Spatial patterns of the indications of acupoints using data mining in classic medical text: a possible visualization of the meridian system. Evid Based Complement Alternat Med 2015;2015:457071. 26539224 10.1155/2015/457071 PMC4619912

[9] Lee IS Lee SH Kim SY Visualization of the meridian system based on biomedical information about acupuncture treatment. Evid Based Complement Alternat Med 2013;2013:872142. 23781270 10.1155/2013/872142 PMC3679759

[10] Lee SH Kim CE Lee IS Network analysis of acupuncture points used in the treatment of low back pain. Evid Based Complement Alternat Med 2013;2013:402180. 23956769 10.1155/2013/402180 PMC3730389

[11] Lee T Jung WM Lee IS Data mining of acupoint characteristics from the classical medical text: DongUiBoGam of Korean Medicine. Evid Based Complement Alternat Med 2014;2014:329563. 25574179 10.1155/2014/329563 PMC4276123

[12] Melzack R The McGill Pain Questionnaire: major properties and scoring methods. Pain 1975;1:277–99. 1235985 10.1016/0304-3959(75)90044-5

[13] Beissner F Marzolff I Investigation of acupuncture sensation patterns under sensory deprivation using a geographic information system. Evid Based Complement Alternat Med 2012;2012:591304. 23243458 10.1155/2012/591304 PMC3518766

[14] Jung WM Shim W Lee T More than DeQi: spatial patterns of acupuncture-induced bodily sensations. Front Neurosci 2016;10:462. 27807402 10.3389/fnins.2016.00462 PMC5069343

[15] Ledberg A Akerman S Roland PE Estimation of the probabilities of 3D clusters in functional brain images. NeuroImage 1998;8:113–28. 9740755 10.1006/nimg.1998.0336

[16] Rong PJ Zhao JJ Gao JH Progress of research on specificity of meridian acupoint efficacy. Chin J Integr Med 2013;19:889–93. 24307308 10.1007/s11655-013-1651-z

[17] Fang J Jin Z Wang Y The salient characteristics of the central effects of acupuncture needling: limbic-paralimbic-neocortical network modulation. Hum Brain Map 2009;30:1196–206. 10.1002/hbm.20583 PMC6871074 18571795

[18] Chen KH Hsiao KY Lin CH Remote effect of lower limb acupuncture on latent myofascial trigger point of upper trapezius muscle: a pilot study. Evid Based Complement Alternat Med 2013;2013:287184. 23710218 10.1155/2013/287184 PMC3655618

[19] Dorsher PT Myofascial referred-pain data provide physiologic evidence of acupuncture meridians. J Pain 2009;10:723–31. 19409857 10.1016/j.jpain.2008.12.010

